# Prediction, validation, and uncertainties of a nation-wide post-fire soil erosion risk assessment in Portugal

**DOI:** 10.1038/s41598-022-07066-x

**Published:** 2022-02-21

**Authors:** J. Parente, A. Girona-García, A. R. Lopes, J. J. Keizer, D. C. S. Vieira

**Affiliations:** 1 Centre for Environmental and Marine Studies (CESAM), Department of Environment and Planning, University of Aveiro, 3810-193 Aveiro, Portugal; 2 European Commission, Joint Research Centre (JRC), Ispra, Italy

**Keywords:** Environmental sciences, Hydrology, Natural hazards, Geomorphology

## Abstract

Wildfires are a recurrent and increasing threat in mainland Portugal, where over 4.5 million hectares of forests and scrublands have burned over the last 38 years. These fire-affected landscapes have suffered an intensification of soil erosion processes, which can negatively affect soil carbon storage, reduce fertility and forest productivity, and can become a source of pollutants. The main objective of the present study is to produce a post-fire soil erosion risk map for the forest and shrubland areas in mainland Portugal and assess its reliability. To this end, the semi-empirical Morgan–Morgan–Finney erosion model was used to assess the potential post-fire soil erosion according to distinct burn severity and climate scenarios, and the accuracy of the predictions was verified by an uncertainty analysis and validated against independent field datasets. The proposed approach successfully allowed mapping post-fire soil erosion in Portugal and identified the areas with higher post-fire erosion risk for past and future climate extremes. The outcomes of this study comprise a set of tools to help forest managers in their decision-making for post-fire emergency stabilization, ensuring the adequate selection of areas for mitigation to minimize the economic and environmental losses caused by fire-enhanced soil erosion.

## Introduction

Soils are one of the most valuable non-renewable Earth resources, containing the largest terrestrial organic carbon stock and supporting natural vegetation as well as human agroforestry systems^1^. In the last decades, due to several causes such as the unsustainable land use and management^2–5^ and climate change^4^, land degradation risk has become one of the most important ecological concerns worldwide^6,7^.

The propensity of Portugal to the occurrence of extreme weather and climate conditions, such as heatwaves and droughts^8,9^, makes it the first southern European country in terms of the number of wildfire events, and the second in terms of the total burned area^10^. Therefore, wildfires constitute one the most significant environmental issues in Portugal, and are frequently considered an important driver of forest soil erosion and land degradation^11,12^. Besides the destruction of vegetation and litter cover that intercepts and protects the soil from direct rainfall impacts, wildfires cause soil heating that, in turn, can reduce soil’s aggregate stability, decrease porosity, and induce water repellency, thus limiting water infiltration and favoring particle detachment^11,13,14^. The combination of these direct wildfire impacts on vegetation and soil negatively affects the forests services, including the water cycle regulation and the soil erosion control^15^. Also, fire-enhanced runoff and erosion responses are well documented to have important off-site consequences such as the contamination of the downstream water bodies by eroded sediments and wildfire ashes^16^, or the occurrence of destructive debris flows^17^ at lower rainfall thresholds.

Consequently, the application of stabilization treatments has been a priority in post-fire forest management, also motivated by the increasing allocation of European funding for the implementation of post-fire erosion mitigation measures^18^. However, the timely implementation of these measures has often been compromised by the lack of methodologies providing a rapid early diagnosis of the areas with higher soil erosion following the wildfire^19^. To address such difficulties, soil erosion modelling arises as a powerful tool, providing crucial information supporting decision-making, both for emergency responses and long-term planning^20^.

In the last twenty years, there has been an increasing number of studies testing different models for post-fire soil erosion predictions^20^ and adapting them to accommodate fire-induced changes in vegetation, soil, and water infiltration. Most of those adaptations are based on the degree of burn severity, motivated by the numerous evidences of its key-role in post-fire soil erosion^11^. One of the most frequently tested models is the revised Morgan-Morgan-Finney model (MMF), especially for burned areas in Portugal^20–22^ and NW Spain^23^. Although MMF has performed well in predicting erosion measured in field studies, there is still an urgent need for a simplified model-based tool to support decision-making in post-fire management. In addition, recent studies^20,24^ stressed that despite advances have been recently made in post-fire soil erosion modelling, model validation against independent datasets and model uncertainty analyses have been limited. The outcomes of uncertainty analyses are vital for forest managers to identify and prioritize intervention areas and make informed decisions^25^ on when and where the often-limited resources can be optimally applied for emergency stabilization^20^.

The present study aims to develop and validate a map of soil erosion risk over the first post-fire year for mainland Portugal (hereafter, Portugal) and assess its uncertainties. This was accomplished using the revised MMF model for the three land cover classes in Portugal that have been most frequently affected by wildfires (eucalyptus, pine, and shrubland)^26^. Two main sources of uncertainty were considered: (i) those related to the post-fire rainfall regime, by using two distinct rainfall datasets (ERA-Interim and ERA5) from the European Centre for Medium-Range Weather Forecasts; (ii) those related with fire severity, using three distinct scenarios of soil burn severity. The reliability of the soil erosion risk map was assessed by comparing the MMF predictions against independent erosion data from several field trials conducted in Portugal during the 2005–2017 period. This study seeks to answer the specific research questions: (i) “Can the revised MMF model be used as a tool to determine post-fire soil erosion risk in Portugal and to support decision-making in post-fire emergency stabilization?”; (ii) “How important are the model input data for accurately predicting post-fire soil erosion risk, and how can this selection affect decision-making in post-fire management?”.

## Materials and methods

**Figure 1:**
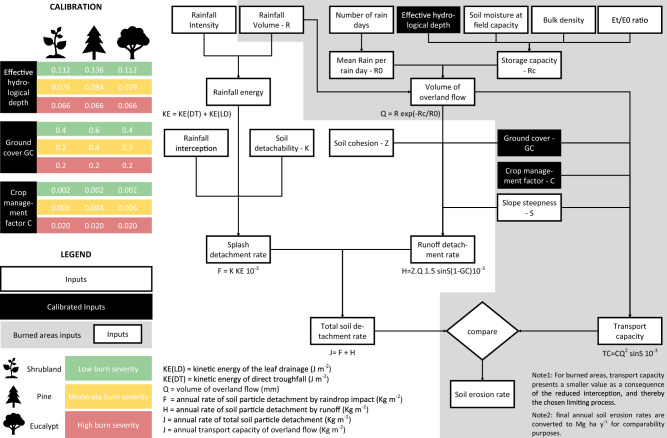
Simplified flow chart of the revised Morgan-Morgan-Finney model, showing the key equations for the different model phases and the parameterization data for different land covers and classes of burn severity.

In this study, we developed a post-fire soil erosion risk map for Portugal using the revised MMF model, by applying a successful field-based parameterization, conducted in previous studies, that addressed different burn severities and land uses^21–23^ (Fig. 1). The development of this map required several steps of data processing, to adjust variables from different sources to the same spatial resolution and to the model input requirements. Afterwards, we computed the revised MMF model for the 38 hydrological years between October 1st 1980 and September 30th 2018, using two different rainfall datasets and for three different soil burn severity scenarios. The inputs for the revised MMF model consisted mostly of spatially distributed data characterizing topography, soil properties, and rainfall regime.

Erosion predictions were classified according to the soil erosion by water classes defined by the Joint Research Center^26^ : [0; 0.1[; [0.1; 0.5[; [0.5; 1[; [1; 2[; [2; 5[; [5; 10[; ≥ 10 Mg ha^−1^ y^−1^. Finally, we performed an uncertainty analysis and validation assessment to the chosen modelling approach, by comparing our results with average field soil erosion measurements and associated local precipitation measurements.

### Study area

**Figure 2:**
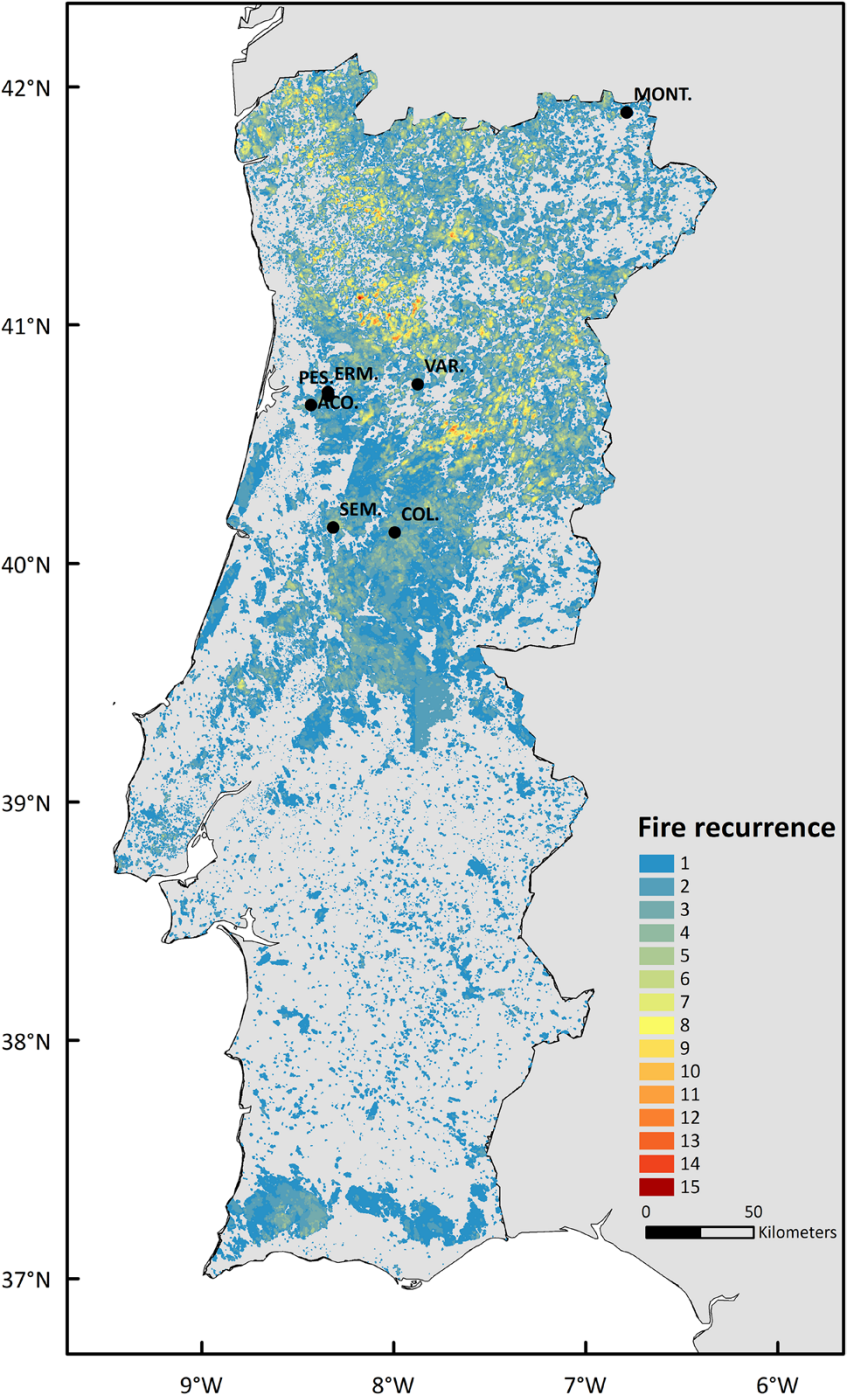
Wildfire recurrence between 1980 and 2018 in Portugal according to the National Mapping Burned Area dataset^28–30^. Points indicate the location of the validation sites defined in Table 2: Montesinho (MONT.), Ermida (ERM.), Pessegueiro do Vouga (PES.), Açores (ACO.), Várzea (VAR.), Semide (SEM.), Colmeal 1 and 2 (COL.).

**Figure 3:**
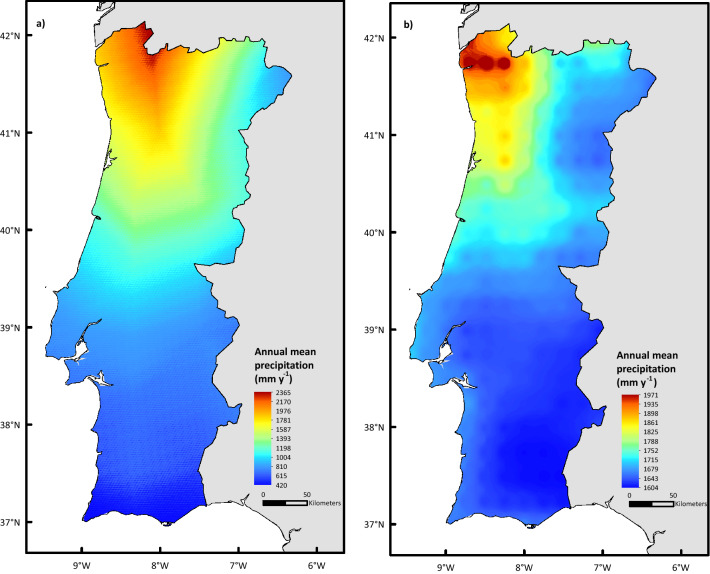
Mean annual precipitation for the 1980–2018 hydrological periods according to the ERA-Interim (**a**) and ERA5 (**b**) datasets. Datasets details available in Table 1.

Portugal is located in Southwestern Europe with a total area ca. 900,000 km^2^ presenting an extensive history of wildfire recurrence (Fig. 2)^9,27^. The mainland area of Portugal presents an altitude ranging from sea level in the western and southern coastal areas to about 2,000 m a.s.l. in the north-central region. It is located in a transition zone between sub-tropical and mid-latitude climates^8^ , where temperate climate prevails with dry and warm summer (Csb) in the north, and with dry and hot summer (Csa) in the south^28^. The type of climate depends on the topographical features of the country and helps explaining the land cover differences among regions (Fig. 3a). Those climate conditions increase the wildfire susceptibility of Portugal, and explains the prominent annual wildfire activity cycle (Fig. 2)^9^.

### Spatially distributed datasets

The gridded global atmospheric reanalysis products of the European Centre for Medium-Range Weather Forecasts ERA-Interim and ERA5 datasets were selected to obtain annual rainfall data (Fig. 3). Both datasets were selected because MMF requires as input a continuous series of daily precipitation data^31,32^.

**Figure 4:**
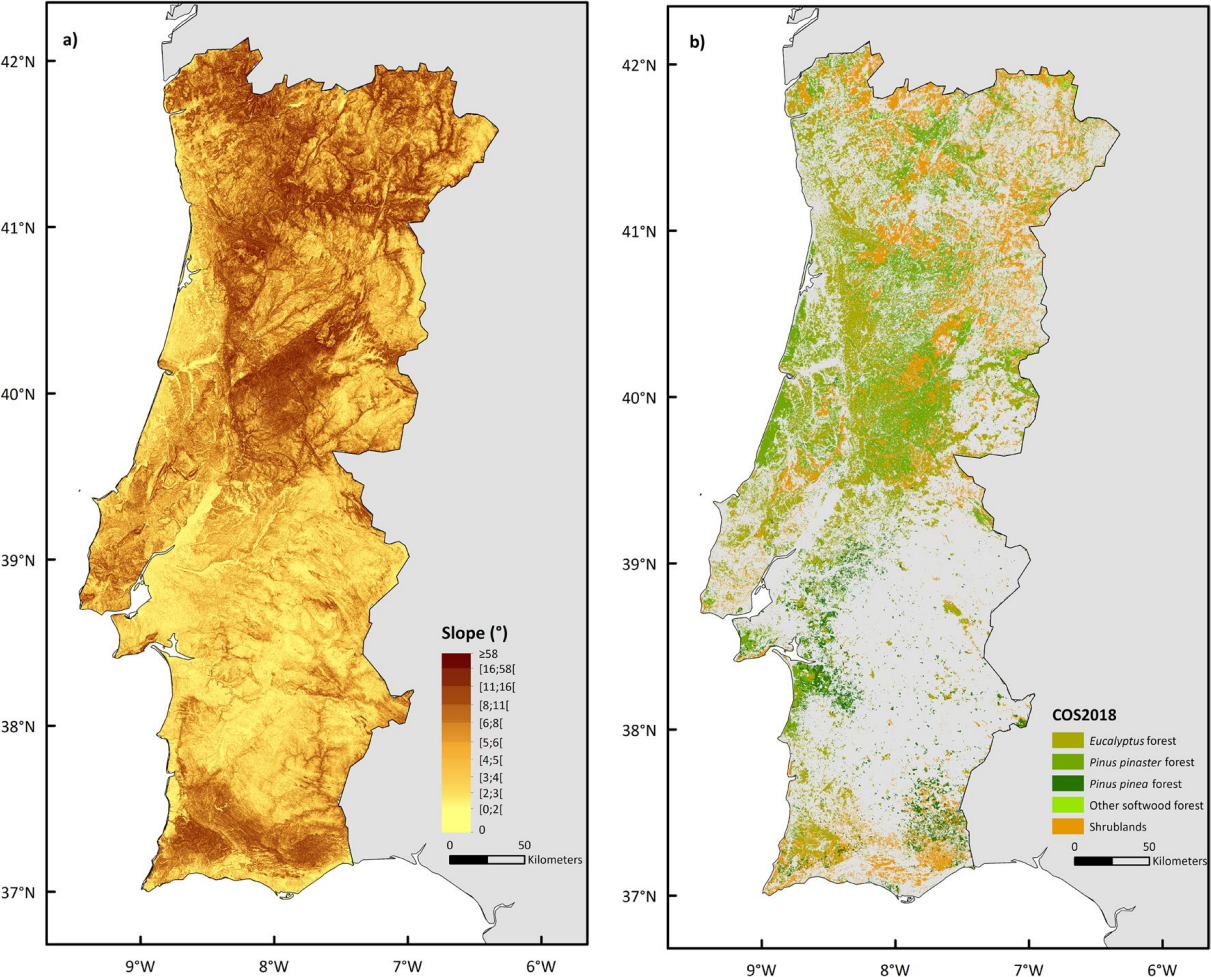
Maps of slope angle (**a**), and of land cover (**b**) for mainland Portugal. Datasets details available in Table 1.

A high-resolution map of slope angle was derived from the Digital Elevation Model that resulted from the Shuttle Radar Topographic Mission (Table 1). The map showed a heterogeneous slope steepness distribution within Portugal, observing the highest values in the north and southernmost regions (Fig. 4a). Soil bulk density was obtained from the European Soil Data Centre dataset^33^, and soil moisture content was obtained from European Space Agency^34–36^(Table 1).

**Table 1:** Spatial datasets used, source of data and original resolution.

Spatial datasets	Source	Resolution
**Rainfall**		
ERA-Interim	European Centre for Medium-Range Weather Forecasts https://apps.ecmwf.int/datasets/data/interim-full-daily/	64 × 10^8^ m^2^
ERA5	European Centre for Medium-Range Weather Forecasts https://cds.climate.copernicus.eu/cdsapp#!/dataset/reanalysis-era5-land	961 × 10^6^ m^2^
**Topography**		
Digital Elevation Model	Shuttle Radar Topographic Mission https://www.fc.up.pt/pessoas/jagoncal/srtm/	625 m^2^
**Soil properties**		
Soil bulk density	European Soil Data Centre^33^ https://esdac.jrc.ec.europa.eu/content/topsoil-physical-properties-europe-based-lucas-topsoil-data	62,500 m^2^
Soil moisture content	European Space Agency^34–36^ https://climate.esa.int/en/projects/soil-moisture/data/	441 × 10^6^ m^2^
**Land cover**		
Land use and occupation 2018 inventory	*Direção Geral do Território* https://www.dgterritorio.gov.pt/Carta-de-Uso-e-Ocupacao-do-Solo-para-2018	400 m^2^

Sub-classes details	Area (ha)	% of total forest
Eucalyptus (*Eucalyptus* spp*.*) forest	928,211	27
Maritime pine *(Pinus pinaster* Ait.) forest	1,020,283	29
Stone pine (*Pinus pinea* L.) forest	211,147	6
Other softwood forests	37,213	1
Shrublands	1,107,546	–

Land cover national information was obtained from the national inventory of 2018 provided by *Direção Geral do Território*. It comprises 83 land cover sub-classes but, given the focus on wildfires, we used five (Table 1). Together, these 5 sub-classes correspond to about 3.3 million ha, which comprise 63% of the main forest types of Portugal and all the shrubland area^37^. The forests dominated by eucalyptus (*Eucalyptus spp.*) and maritime pine *(Pinus pinaster* Ait.) are mostly located in Central Portugal (Fig. 4b), while the stone pine (*Pinus pinea* L.) forests are mainly located immediately at the top of south region (Fig. 4b). The shrubland areas are the dominant land cover in the northeastern and southernmost regions of Portugal.

The soil bulk density, soil moisture content, and ERA5 datasets needed to be scaled down to fit the 25 m grid dimension (Table 1). To this end, the Inverse-Distance Weighting method (IDW) was used, which is a spatial interpolation deterministic model that was computed using R gstat package^38^.

### Reference soil erosion and local precipitation

MMF performance as well as the global rainfall datasets were validated against published field observations. To this end, the SCOPUS dataset was searched to compile all peer-reviewed articles in English in international journals that reported annual post-fire erosion rates in Portugal. The resulting 11 articles included a total of 10 field sites with micro-plot to swale-scale erosion data on 3 to 18 plots per site, giving a total of 20 years of study (Table 2). The articles also reported the relevant rainfall data for all 10 sites. There was a strong bias in the geographical location of the field sites, the bulk of them being located in north-central Portugal. This bias, however, largely coincides with the strong prevalence of burned areas in north-central Portugal over the past decades (Fig. 2), and also reflects the lack of post-fire erosion studies in the other parts of the country.

**Table 2:** Description of the field sites and experimental designs used for validation of post-fire soil erosion predictions by MMF.

Location, municipality^reference^	Coordinates	Burn severity^a^	Monitoring years	Nr. erosion plots	Plot size (m^2^)	Annual rainfall (mm)
Açores, Albergaria‐a‐Velha^52^	40°40′46·62″N 8°26′54·80″W	Moderate	2005 – 2006	8	0.25	1048 – 1608
Colmeal, Góis^14^	40°08′42″N 7°59′16″W	Moderate – High	2008 – 2010	3^b^	10	
Colmeal, Góis^22,40^	40°08′48"N 7°59′50"W	Moderate	2008 – 2012	16	0.25 – 0.5	833 – 1534
Pessegueiro do Vouga, Sever do Vouga^53^	40° 43′05″N 8° 21′15″W	Moderate – High	2007	6^b^	16	1546
Ermida, Sever do Vouga^54^	40°44′05″N 8°21′18″W	Moderate – High	2010	4^b^	0.25	1475
Ermida, Sever do Vouga^55^	40°44′05″N 8°21′18″W	Moderate	2010	3^b^	100	1475
Montesinho, Bragança^56^	41°53′57″N 6°40′55″W	Low	2011	6	4	545
Várzea, Viseu^57^	40°45′58″N, 7°51′35″W	Moderate	2012 – 2013	18	0.25	1289–1628
Semide, Miranda do Corvo^58^	40°09′57"N 8°19′31"W	Moderate – High	2015 – 2016	3	16	1291
Semide, Miranda do Corvo^59^	40°09′57"N 8°19′31"W	Moderate – High	2015 – 2017	3	1000	1291

(a) Authors classification and/or according to Vega et al*.*^60^ when unavailable;

(b) Only control plots used, since the original study tested mitigation measures.

### Post-fire soil erosion predictions by the MMF model

The MMF model predicts annual runoff and associated soil losses at slope scale^22^, separating the processes in a water phase and a sediment phase^39^ (Fig. 1). The first phase simulates overland flow generation as well as detachment of soil particles by rainfall and runoff, while the second phase models the transport capacity of the overland flow to transport the detached soil particles^22,39^.

In this study, the MMF model was applied to predict annual soil losses for a 38-year period, considering three scenarios of soil burn severity (low, moderate, and high). The maximum annual erosion rates predicted over this period of time were then used for mapping post-fire erosion risk. The MMF parameters and associated model input datasets were the following:

1. Annual rainfall (mm yr^−1^) and days with rain: derived from the above-mentioned global rainfall datasets;
2. Soil parameters: bulk density (BD, g cm^−3^), soil detachability index (K, g J^−1^), and soil surface cohesion (COH, kPa), were estimated from LUCAS dataset^32^. The effective hydrological depth of soil (EHD, m) was calibrated with field data^14,40^ , and previous MMF applications in recently burned areas in north-central Portugal^21–23^ (Fig. 1);
3. Landform: represented as slope steepness (S, °);
4. Land cover parameters: rainfall interception (A) and canopy cover (CC, %), plant height (PH, m) were approximated to 0 given the wildfire effects. The ratio of actual (Et, mm) to potential (E0, mm) evapotranspiration, crop cover management factor (C), and ground cover (GC, %) and estimated from previous MMF applications in recently burned areas in north-central Portugal^21–23^ (Fig. 1);
5. Maximum soil moisture at storage capacity: estimated from soil moisture dataset.

### MMF uncertainty analysis and validation

The parametric uncertainty analysis of the MMF erosion predictions was performed with respect to the rainfall input data. To this end, the predictions resulting from the ERA-Interim and ERA5 datasets were compared in terms of total predicted soil losses, magnitude, and spatial discrepancies. In addition, MMF performance was assessed by comparing the predicted and observed post-fire erosion rates for the 10 field sites listed in Table 2. This was done using the rainfall data from the two global rainfall datasets and from the field studies. To quantify MMF performance, the following four indicators were used in line with previous studies^21,22,41^:

1. Root mean square error (RMSE): represents the measure of the predicted variation to observed data;
2. Nash–Sutcliffe efficiency (NSE): expresses the magnitude of the residual variance relative to the variance in the measured data;
3. Coefficient of determination (R^2^): explains how well a model performs when replicating the observed outcomes;
4. Percent bias (PBIAS): expresses the degree to which predicted values underestimate (negative PBIAS) or overestimate (positive PBIAS) the observed values.

It should be noted, however, that such metrics are more frequently used within continuous catchment hydrology modelling approaches than for spatially distributed predictions^39^. Despite those limitations, such methodology is still the most suitable one to assess model performance and adaptation.

## Results

### Post-fire soil erosion risk mapping

**Figure 5:**
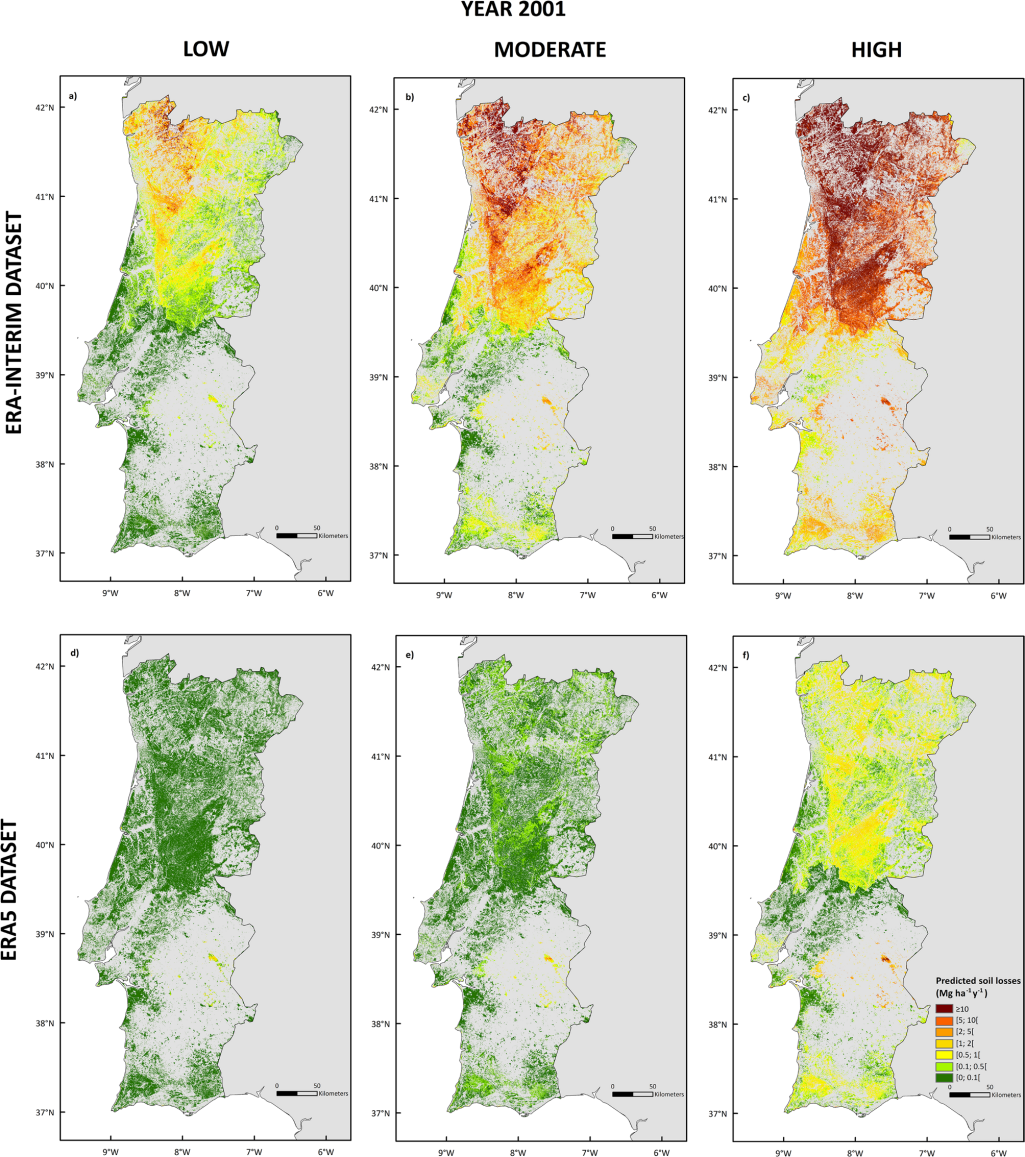
Predicted soil loss risk (Mg ha^−1^ y^−1^) for each burn severity scenario (low, moderate, and high) in the year 2001, according to the ERA-Interim (**a**–**c**, respectively) and ERA5 (**d**–**f**, respectively) rainfall datasets.

The nation-wide application of the MMF model to Portugal revealed substantial differences in terms of predicted soil erosion amounts based on the rainfall dataset choice (Fig. 5). For the entire studied period (1980 to 2018), the highest soil erosion predictions were found for the year 2001, amounting to a total of 6,854,429 and 210,317 Mg y^−1^ of soil loss under severe burning conditions for the ERA-Interim and ERA5 rainfall datasets, respectively. The corresponding post-fire soil erosion risk map revealed that, according to ERA-Interim, 55.7% of the studied area would be at serious risk of soil losses in case of a severe wildfire (> 10 Mg ha^−1^ yr^−1^), while for ERA5 that erosion risk class would be present in only 0.1% of the study areas.

Nevertheless, in both cases the highest post-fire soil erosion risk classes are predominantly found in North-Central Portugal, coinciding with a greater distribution of steep slopes (Fig. 4) and high rainfall amounts (Fig. 3), over areas with shrublands and eucalyptus as dominant vegetation cover (Fig. 4).

When considering the effect of burn severity in our modelling predictions, it is noteworthy that soil burn severity dramatically increases sediment losses, being this aspect more evident for ERA-Interim than for ERA5 (Fig. 5). For low burn severity, predicted soil losses are mostly (99.9%) below the 10 Mg ha^−1^ yr^−1^ threshold for ERA-Interim, while for ERA5 this limit decreases to 1 Mg ha^−1^ yr^−1^. For moderate burn severity conditions, the areas with predicted post-fire erosion risk above the threshold of 10 Mg ha^−1^ yr^−1^ substantially increased for the North of Portugal using the ERA-Interim dataset, while for ERA5 these rates are still below. For high burn severity, 14% of the target area presents a severe risk of post-fire soil erosion, with rates above 50 Mg ha^−1^ yr^−1^ for the ERA-Interim dataset, and predominating 1 to 2 Mg ha^−1^ yr^−1^ classes for the ERA5 dataset.

### Model uncertainty analysis and validation

**Figure 6:**
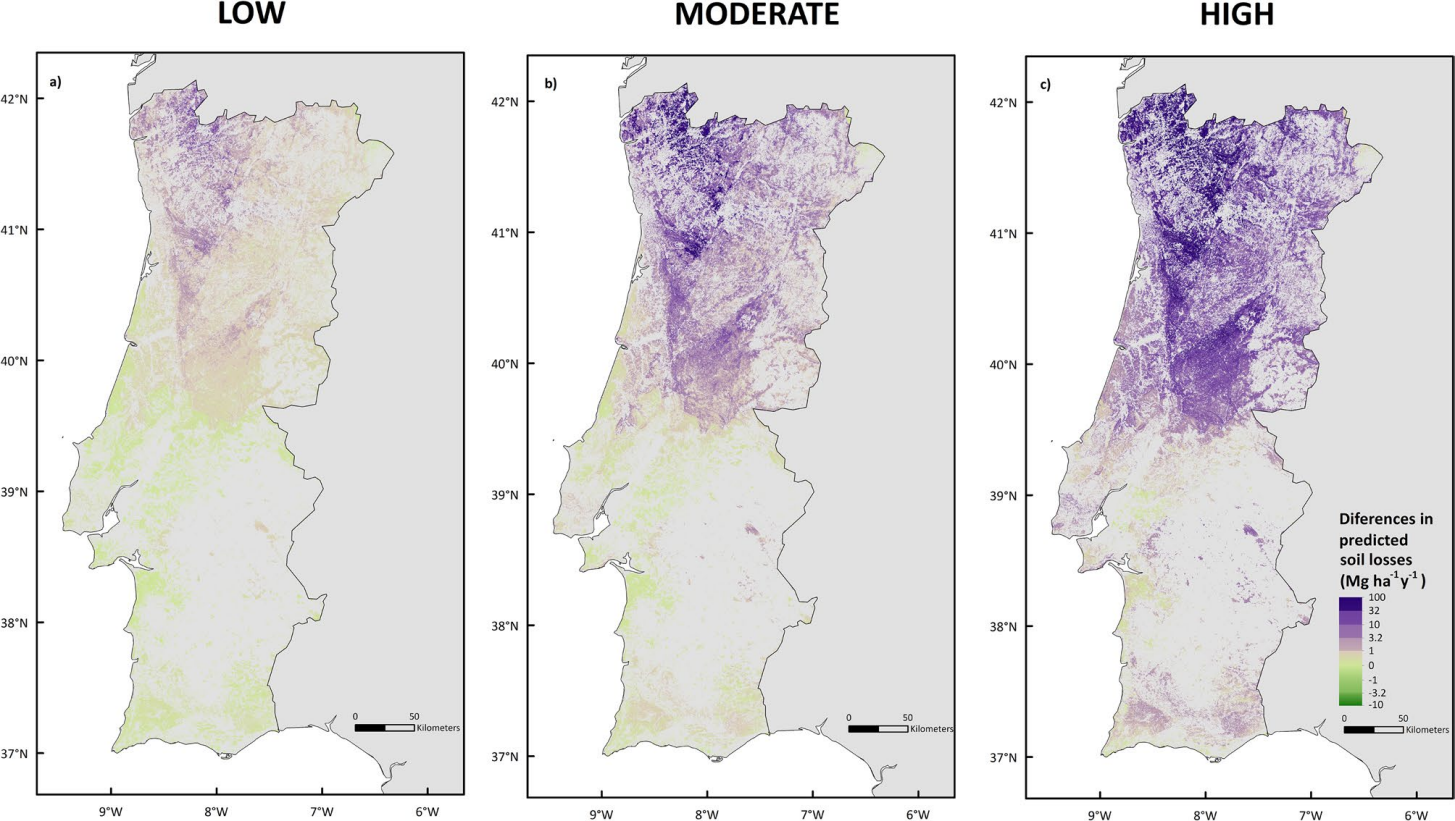
Maps of the differences between the soil losses (Mg ha^−1^ y^−1^) predicted for the year 2001 comparing the ERA-Interim and ERA5 rainfall datasets for three burn severity scenarios (low, moderate, and high), according with quantile measures.

Our results show that, for the high burn severity scenario, soil erosion predictions obtained using the ERA-Interim data set are two orders of magnitude higher than those using the ERA5 data set. Despite the fact that the spatial distribution of rainfall of both datasets presents slight differences (Fig. 4), no major spatial discrepancies were found when predicting post-fire soil erosion (Fig. 6); being the ones resulting from ERA-Interim consistently higher than those from ERA5 because of its almost consistently higher annual rainfall amounts, with the exception of few locations (0.002%).

**Figure 7:**
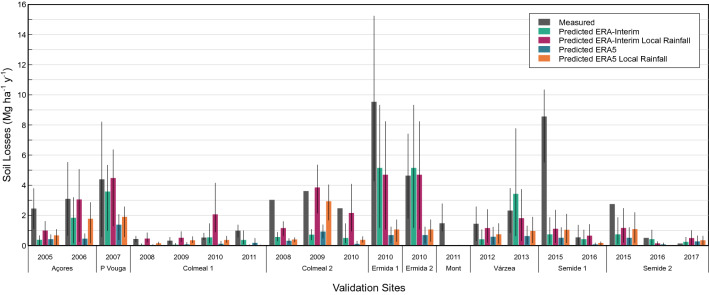
Measured and predicted soil losses (Mg ha^−1^ y^−1^) for reference soil erosion sites (Table 2) according to ERA-Interim and ERA5 datasets, and local rainfall measurements.

**Figure 8:**
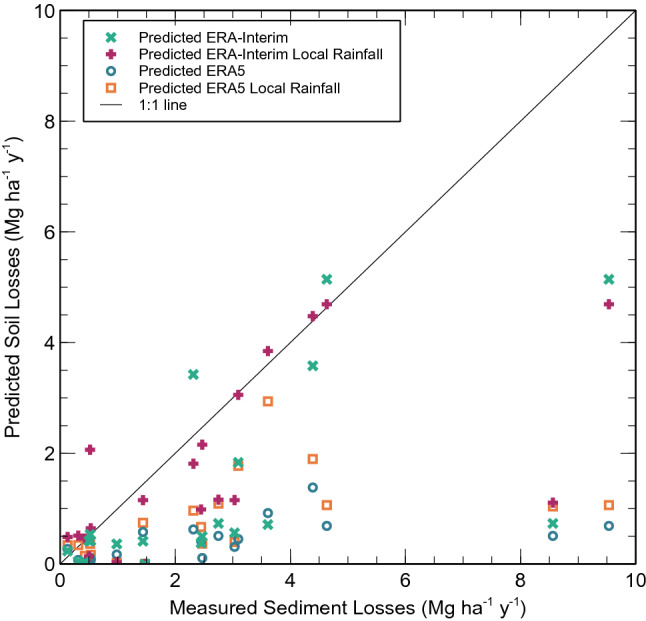
Comparison between measured and predicted soil losses (Mg ha^−1^ y^−1^) for reference soil erosion sites (Table 2) according to ERA-Interim and ERA5 datasets, and local rainfall measurements.

Overall, the MMF predictions for the field sites presented a poor agreement with the observed average post-fire erosion rates, which could be related to their high variability (Table 3; Fig. 7). In terms of all four indicators, MMF performance was not satisfactory for any of the four rainfall datasets according to the thresholds proposed in Moriasi et al*.*^41^. Nonetheless, MMF performance did differ noticeably between the four data sets. Overall, it was best when using the local annual rainfall data combined with the ERA-interim data (number of raining days), and worst when using the ERA5 dataset. Furthermore, the use of local rainfall data generally improved model performance for the two global rainfall datasets, except in terms of R^2^ in the case of the ERA5 dataset. Finally, MMF performance was markedly better for the ERA-Interim than for the ERA5 dataset, in terms of all four indicators. In this way, the ERA5 dataset led to a higher underestimation of observed erosion rates than ERA-Interim, as shown by the negative PBIAS (Table 3) and Fig. 8.

**Table 3:** MMF performance metrics for the prediction of annual post-fire erosion rates observed at 11 field sites for four different rainfall datasets.

Model performance indicators	ERA-Interim	ERA-Interim and local rain	ERA5	ERA5 and local rain
NSE	0.09	0.26	−0.67	−0.36
PBIAS (%)	−52.6	−34.8	−85.0	−71.0
RMSE (Mg ha^−1^ yr^−1^)	2.40	2.16	3.25	2.94
R^2^	0.40	0.40	0.33	0.24

*NSE* Nash–sutcliffe efficiency,* PBIAS* percent bias,* RMSE* root mean square error,* R*^2^ coefficient of determination.

## Discussion

This study aims to produce a soil erosion risk map for the wildfire-prone vegetated areas in Portugal^26^ by using the revised MMF model. This model has been previously calibrated for Portugal^21,22^ and NW Spain^23^ and has demonstrated great capacity to simulate annually and seasonally the hydrological and erosive response in recently burned forest areas^21,22,42^.

**Figure 9:**
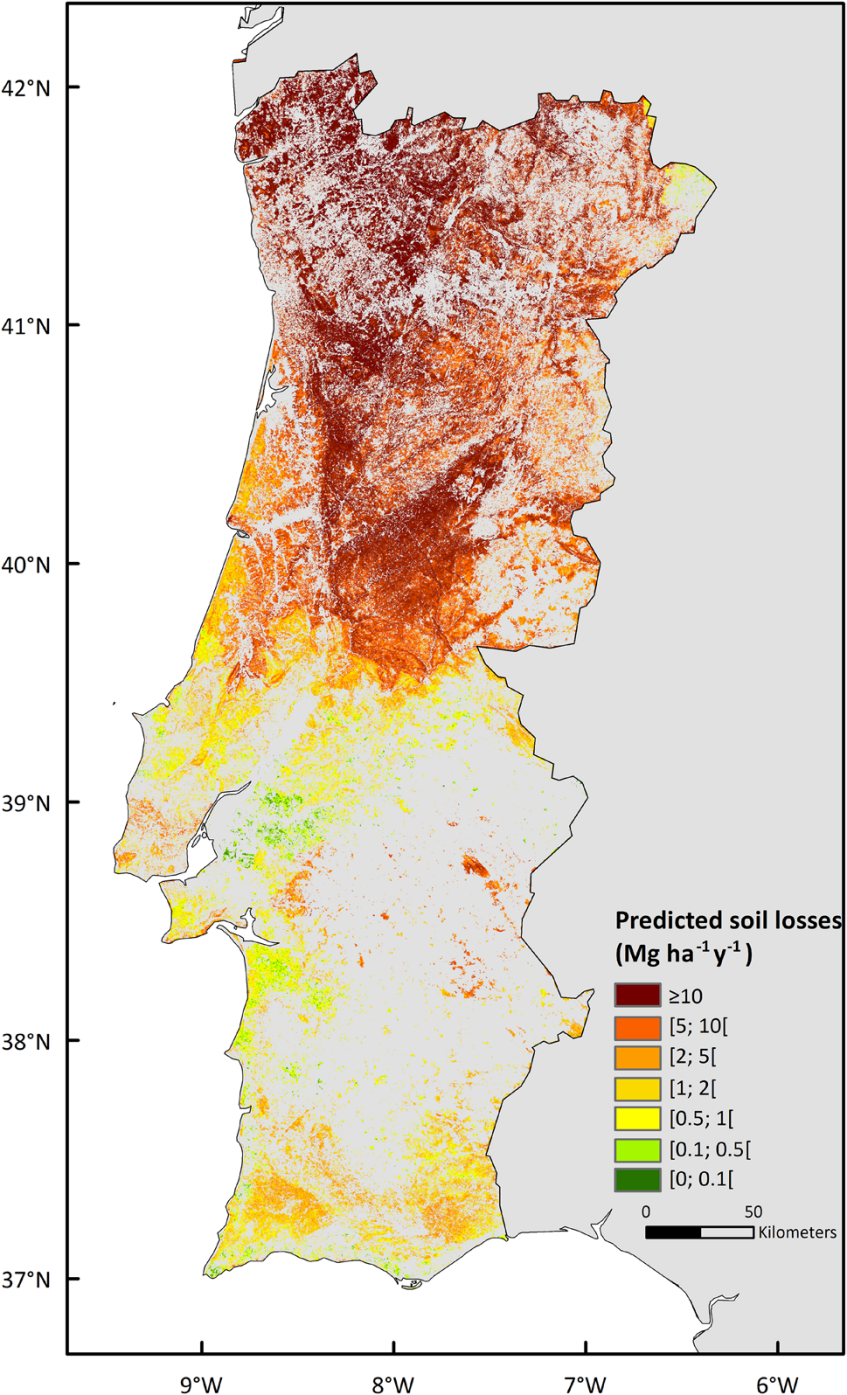
Post-fire soil erosion risk map for eucalypt, pine and shrubland areas in mainland Portugal.

As a final outcome of this study, such post-fire soil erosion risk map for Portugal was possible to be created with the available data, although several important uncertainties were identified during its development. Nevertheless, given the uncertainty analysis and the model performance assessment made with the local validation sites, the high severity scenario obtained using ERA-Interim was selected for the nation-wide post-fire erosion risk map, as its estimates agreed best with the field observations (Fig. 9).

Despite areas at risk of extreme post-fire soil erosion rates (> 10 Mg ha^−1^ y^−1^) were identified in both north and south of Portugal, the north-central part of the country showed a much higher density of locations with such risk (Fig. 5). This can be justified by the greater eucalypt, pine and shrubland cover areas (Fig. 3), and the highest distribution of steep slopes at these locations. Such result also agrees with previous studies, and despite Panagos et al*.*^43^ used a different rainfall input in the form of rainfall erosivity, their soil erosion risk obtained for Europe by the Revised Universal Loss Equation model (RUSLE) presents a similar spatial pattern to ours^44,45^.

This implies that in order to prevent post-fire soil erosion in recently burned areas, the decision-makers and land managers of the north-central region areas of Portugal need to be aware of such risks. Especially considering that those high-risk areas are recurrently affected by wildfires as the historical data shows (Fig. 2), and that these same locations also provide important ecosystems services to society such as the maintenance of water quality and flood control, which can be severely affected by post-fire soil erosion^16,46,47^.

This post-fire soil erosion risk map (Fig. 9) will help managers in the decision-making process after wildfires, allowing the identification of high-risk areas and thus, of priority for intervention, ensuring the most efficient implementation of post-fire mitigation measures^19^ . It should be highlighted however, that the transformation of the predictions obtained in this study to an easily accessible tool for post-fire management would further improve decision-making in these high-risk areas, by considering the various scenarios of severity and the specificities of each affected area in a dynamic way.

A parametric uncertainty analysis was performed to be used as a guideline in the decision-making process when the purpose is to consider and apply, on time, the proper soil erosion mitigation measures after wildfire. Taking this in mind, the methodology used to create the predictions was practical, scientifically defensible, and robust enough to be applicable. For validation matters, several field data were considered as the best estimation possible of the post-fire soil erosion rates.

The differences between the predictions from ERA-Interim and ERA5 (Fig. 6) underline that different precipitation datasets can contain significantly different spatiotemporal information, which might lead to contrasting soil erosion estimations. Also, the authors acknowledge that these discrepancies might be from the limitations of IDW used to scaled down ERA5, which is in spite of being simple, fast, and easy to compute and to interpret^48^, has some limitations such as: (i) weighting parameters are chosen a priori, and not empirically; (ii) the variances of predicted values in unsampled locations cannot be estimated; and (iii) the IDW standard application assumes that the distance-decay relationship is constant through space. In this respect, it is important to underline the good agreement between total annual mean precipitation results obtained in this study with ERA-Interim for 1980–2018 period (Fig. 4) and the maps published in the Iberian climate Atlas produced with data observed in a large set of weather stations, although for a slightly different (1971–2000) period^49^.

Finally, the authors acknowledge that biases of the reanalysis may affect the agreement between the soil erosion predictions and soil erosion. In addition to that, some limitations were also found concerning the available methodologies to assess model performance, especially for validation purposes. By using Moriasi et al*.*^41^metrics, only average soil erosion measurements were used, which did not account for the high variability of field results. Notwithstanding, ERA-Interim seems to be the most advisable reanalysis product because it produced the best results (Fig. 7), despite the underestimations found when compared with the field validation data. Which is in line with Belo-Pereira et al*.*^50^, who have demonstrated that ERA-Interim underestimates precipitation in the rainiest months in Western Iberia and tends to overestimate it in NE and SE regions.

It is therefore advisable that authors consider more than one source of data when creating risk maps to account for additional uncertainties in the predictions; in addition to considering local data when available to better evaluate post-fire soil erosion assessment, as illustrated by the two main sources of uncertainties highlighted in this study (severity and rainfall). Those considerations would eventually translate into more detailed and accurate risk maps and thus, better-informed land management decisions^24^. Identifying all the possible outcomes can help land managers and policy makers to understand that there is always some level of uncertainties associated to post-fire responses, and that those uncertainties should be included in the decision making. In addition to that, it should be highlighted that additional sources of uncertainties other than those pointed out in this study can also be included in such risk assessment. Aspects such as future climate demands, assessment scale, land management operations, or even the inclusion of land uses different to those identified in this study can improve the understanding of the risk at hand; whether the focus is the mitigation of the impacts of the current wildfire, or the prevention of future post-fire impacts, but also, if such reflections are made at local scale or at a political level.

Moreover, it should be highlighted that wildfire occurrence is not exclusive from the Portuguese territory and corresponds to a global environmental problem^51^, and therefore the application of such approach should be considered at the European or Global scale.

## Conclusions

This study produced a soil erosion risk map for the most fire-prone vegetated areas in Portugal (eucalyptus, pine and shrubland), based on the revised Morgan–Morgan–Finney model predictions and validated using local post-fire field data, which, to the best of our knowledge, has never been done for Portugal.

The main conclusions drawn while developing this study can be summarized as follows:

- the highest post-fire soil erosion risk is estimated in north-central Portugal, and land managers should be aware of it, especially given the national wildfire recurrence history;
- the development of risk maps should consider different data sources to account for uncertainties in their predictions, thus creating better decision-making products;
- the validation of soil erosion estimations with field data is important to evaluate the robustness of model predictions, compare distinct scenarios, and understand which data sources are the most suitable for model application.

The authors believe this modelled map could be of great use for land managers in the post-fire decision-making process in Portugal, allowing the early identification of critical areas for the implementation of emergency stabilization measures, and such work should be expanded outside the Portuguese territory.
